# Laboratory Performance of Heat-Assisted Fly Ash-Based Geopolymer Concrete as a Potential Thin Protective Layer for Asphalt Pavements

**DOI:** 10.3390/ma19153170

**Published:** 2026-07-24

**Authors:** Krzysztof Granatyr, Michał Bołtryk, Katarzyna Kalinowska-Wichrowska, Edyta Pawluczuk

**Affiliations:** Faculty of Civil Engineering and Environmental Sciences, Bialystok University of Technology, Wiejska 45E, 15-351 Bialystok, Poland; m.boltryk@pb.edu.pl (M.B.); k.kalinowska@pb.edu.pl (K.K.-W.); e.pawluczuk@pb.edu.pl (E.P.)

**Keywords:** geopolymer concrete, alkali-activated materials, fly ash, asphalt–geopolymer composite, heat-assisted curing, thin protective layer, wheel tracking, fatigue, skid resistance, freeze–thaw resistance

## Abstract

This study presents a laboratory-scale assessment of heat-assisted fly ash-based geopolymer concrete as a potential thin protective layer in one asphalt–geopolymer pavement configuration. The programme comprised water penetration under pressure, abrasion, initial skid resistance, wheel tracking, four-point-bending fatigue, mechanical strength, freeze–thaw response, de-icing-salt scaling, thermal characterization, and qualitative scanning electron microscopy. The selected geopolymer reached mean flexural, compressive, and splitting tensile strengths of 10.756, 61.058, and 3.797 MPa, respectively. Final rut depths were 1.57 mm after 7 days and 0.72 mm after 28 days, with corresponding WTSAIR values of 0.020296 and 0.014238 mm per 10^3^ cycles. After 10^6^ cycles at 10 Hz, 72–85% of the initial stiffness modulus remained across the tested strain levels. Mean de-icing-salt scaling was 0.500 kg/m^2^ after 28 days and 0.995 kg/m^2^ after 56 days. Standalone geopolymer specimens underwent full-depth water penetration, whereas no leakage through the asphalt layer was observed in the intact layered specimen during the 5 bar, 72 h test. This system-level observation supports functional tightness only under the tested intact condition and does not establish intrinsic material impermeability or long-term interface durability. Interpretation is limited to this laboratory-scale configuration: the 90 °C heat-assisted curing protocol limits transfer to conventional in situ paving; no ambient- or standard-cured control and no same-condition conventional overlay control were included; and only one composite geometry was evaluated. The findings therefore define application boundaries for further validation rather than a field-ready specification or proof of comparative superiority.

## 1. Introduction

Road surface layers must retain load-carrying capacity, texture, and continuity while exposed to traffic, water, de-icing agents, and temperature variation. In multilayer asphalt pavements, permanent deformation, fatigue cracking, and loss of interlayer bond can accelerate structural deterioration and increase maintenance demand [1,2,3]. Protective surface systems are therefore of interest for locations such as intersections, bus bays, braking zones, and slow lanes, where concentrated traffic effects and rutting risk can be high.

Geopolymer concrete is produced by alkaline activation of aluminosilicate precursors such as fly ash, slag, or metakaolin. Its performance depends on precursor chemistry, activator composition, liquid-to-precursor ratio, aggregate skeleton, compaction, and curing history [4,5,6,7,8,9,10,11,12]. Fly ash-based systems can provide substantial strength and a route for using an industrial by-product. Their environmental profile is not automatically favourable, however, because sodium hydroxide and sodium silicate production, transport, raw material variability, and curing energy can materially affect the balance [10,13]. Environmental and cost interpretations must therefore remain formulation- and process-specific and require life-cycle assessment and life-cycle costing.

Using geopolymer concrete as a thin upper layer on asphalt introduces an additional design boundary: system performance depends on both the geopolymer and the asphalt–geopolymer interface. Earlier published work examined composition and initial interlayer response [14,15] and provided the rationale for selecting the material pair used here. That evidence does not replace direct measurements of bond retention after fatigue, freeze–thaw exposure, thermal cycling, prolonged water ingress, or combined ageing.

The present paper evaluates one selected heat-assisted fly ash-based formulation as a laboratory protective-layer system. The experimental programme comprises water penetration under pressure, abrasion, initial skid resistance, wheel tracking, four-point-bending fatigue, mechanical strength, freeze–thaw response, de-icing-salt scaling, thermal properties, and qualitative SEM observations. The study is not a full-factorial mixture-optimization programme and does not provide a same-condition comparison with conventional asphalt, cement concrete, or thin-whitetopping overlays.

Accordingly, the objectives were to (i) quantify the laboratory response of the selected geopolymer and asphalt–geopolymer specimens; (ii) distinguish standalone material behaviour from the behaviour of the intact layered system; (iii) explain the basis for selecting the reference formulation; and (iv) define the application boundaries within which engineering implications can be drawn. The work is a configuration-specific laboratory assessment, not a field validation or a demonstration of superiority over established pavement systems.

## 2. Materials and Methods

### 2.1. Constituent Materials

#### 2.1.1. Fly Ash

**Figure 1:**
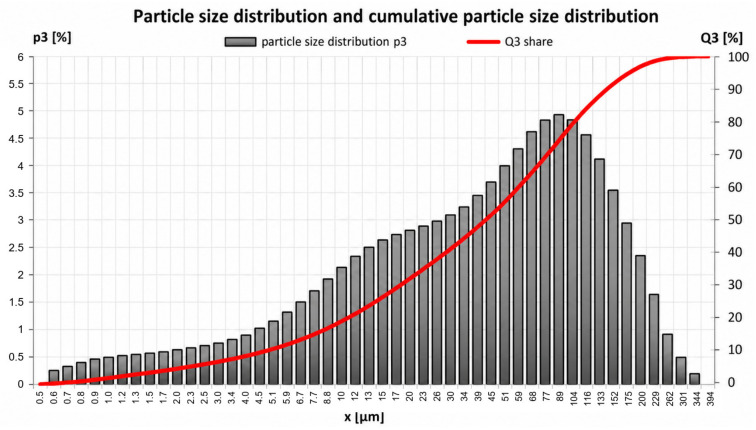
Particle-size distribution and cumulative particle-size distribution of the fly ash.

Fly ash from the coal-fired power plant in Ostrołęka, Poland, was used as the aluminosilicate precursor. Its reported oxide composition is given in Table 1, and the particle-size distribution is shown in Figure 1. The loss on ignition was 4.37%.

#### 2.1.2. Alkaline Activator

The alkaline activator was prepared from sodium silicate (Na_2_SiO_3_) and an aqueous NaOH solution. The sodium silicate-to-NaOH solution mass ratio was 2.5:1. The reference mixture used 12 M NaOH; 8 M and 10 M variants were additionally prepared for the water penetration and skid resistance comparisons.

#### 2.1.3. Asphalt Concrete

The mineral–asphalt mixture was designed as AC16W 35/50 using MASA software developed by Masbit Co. Adam Ruciński—Poland and the technical requirements cited in WT-2 [2]. The mixture used limestone filler, glacial sand and crushed glacial aggregate, and 35/50 paving-grade bitumen conforming to PN-EN 12591:2010 [16]. Its composition is given in Table 2. The reported bulk density was 2321.75 kg/m^3^ and the air-void content was 6.56%.

### 2.2. Reference Mixture Selection

The reference mixture was selected from earlier published mixture development studies [14,15], rather than from the present functional tests alone. Those studies examined NaOH concentrations of 8, 10, and 12 M; mortar-content levels of 45%, 55%, and 65%; and different protective-layer geometries together with strength and initial interlayer response. The selected 12 M, 55% mortar-content formulation represented a multi-criteria compromise. A 65% mortar-content variant produced a higher interlayer response in some conditions, whereas the 55% level provided higher compressive strength while retaining the interlayer response used in the earlier selection. The formulation therefore did not maximize every individual response and is not presented as a universal optimum. The present programme did not independently optimize activator composition or field layer thickness.

**Figure 2:**
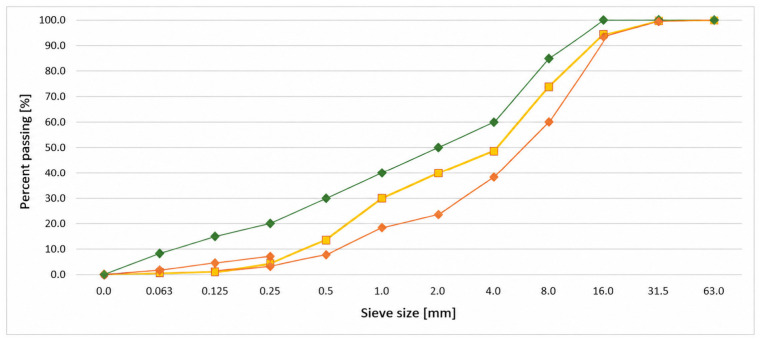
Aggregate grading curve of the reference geopolymer concrete with the roller-compacted-concrete grading limits used in mixture development. Green—upper grain size limit; Orange—lower grain size limit; Yellow—grain size curve.

The fly ash and activator contents were 400 and 200 kg/m^3^, respectively, giving an activator-to-fly-ash mass ratio of 0.50. Aggregate grading was adapted from roller-compacted-concrete design guidance [17]. A maximum aggregate size of 11 mm was used in the geopolymer, compared with 16 mm in the asphalt concrete. Basalt and melaphyre were used as the coarse aggregates in accordance with the material-selection basis described in Ref. [18]. The resulting composition is given in Table 3, and its grading is shown in Figure 2.

### 2.3. Specimen Preparation and Curing

**Figure 3:**
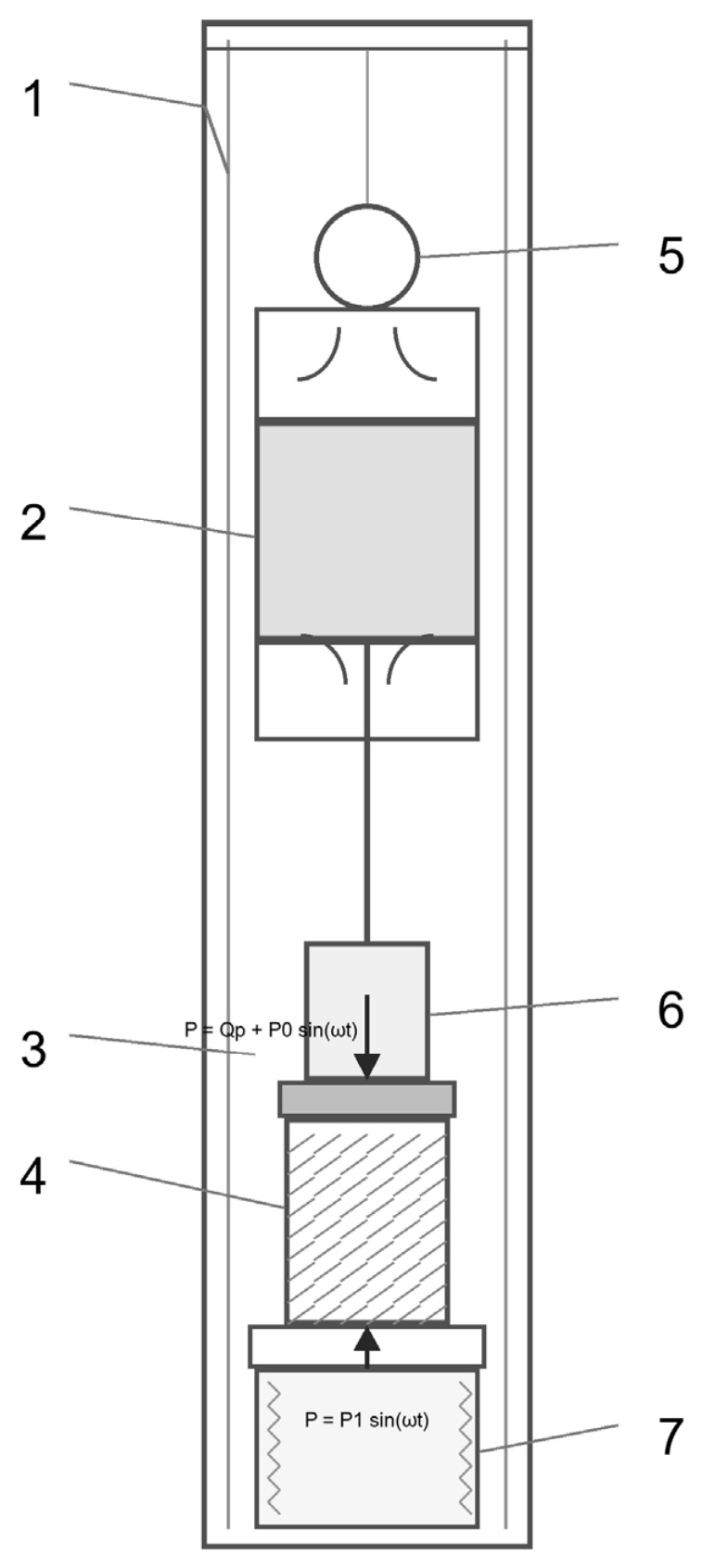
Vibropress used to form the geopolymer specimens: Qp, compaction force; P_0_, excitation force of the upper vibrator; P_1_, excitation force of the lower vibrator; 1, guides; 2, inertial load; 3, extension frame; 4, mould; 5, upper vibrator; 6, pressing piston; 7, lower vibrator.

The aggregate and fly ash were dry mixed before the alkaline activator was added. Mixing continued until a visually homogeneous mixture was obtained. Cubes measuring 100 × 100 × 100 mm were used for water penetration, abrasion, splitting tensile strength, and freeze–thaw tests. Compaction was performed in a vibropress in two stages: 30 s with the lower vibrator followed by 60 s with simultaneous upper-press and lower-vibrator operation (Figure 3).

**Figure 4:**
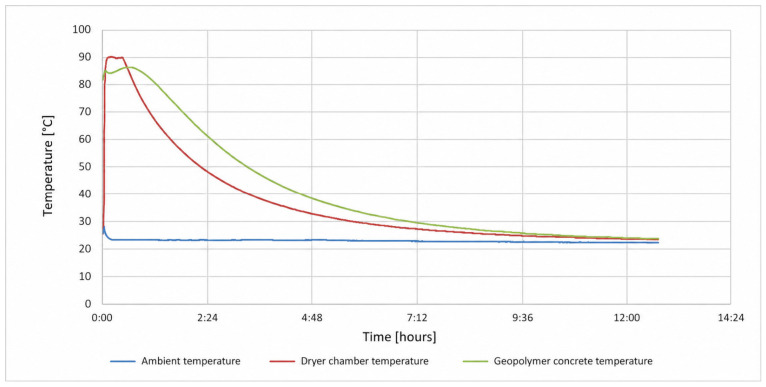
Temperature decrease during the controlled 90 °C hot-start and passive-cooling protocol.

The steel moulds were preheated to 90 °C. After casting, the moulds were placed in a laboratory dryer initially at 90 °C; the dryer was switched off after closing and the specimens cooled passively. Thus, the protocol was a controlled 90 °C hot start followed by slow cooling, not continuous isothermal exposure at 90 °C. The measured cooling profile is shown in Figure 4. After demoulding, the specimens were stored at a relative humidity above 95% until testing.

For asphalt–geopolymer cubes, hot asphalt concrete was compacted to half the mould height, after which the remaining volume was filled with geopolymer concrete and compacted. Beams for flexural and fatigue testing measured 100 × 100 × 500 mm at casting; the fatigue specimens used for 4PB-PR testing were subsequently prepared to 55 × 55 × 400 mm. The geopolymer beams were compacted with a vibrating hammer in accordance with PN-EN 13286-51:2005 [19]. Slabs for skid and wheel tracking tests measured 300 × 400 × 100 mm and were compacted in a semi-roller compactor, with the asphalt layer placed first and the geopolymer layer applied second.

No standard-cured or ambient-cured geopolymer control group was included. The curing protocol was selected for repeatable laboratory geopolymerization and materially limits direct extrapolation to in situ road construction.

### 2.4. Test Programme

The test methods and specimen types are summarized in Table 4. Water pressure was applied from the geopolymer side of the layered specimen. Wheel tracking was conducted in air at 65 °C. Four-point-bending fatigue was run at one loading frequency, 10 Hz, at strain levels of 70, 90, 110, and 130 µm/m for 10^6^ cycles. Consequently, the fatigue results characterize the specified laboratory condition rather than the full frequency spectrum of vehicle loading.

### 2.5. Reporting and Scope of Comparison

Where replicate-level data were available, sample count, range, and standard deviation are reported. Wheel tracking and fatigue comparisons are descriptive and are evaluated against the cited technical benchmarks; they are not inferential comparisons with an independently tested conventional control. The literature values in the thermal discussion are contextual benchmarks rather than same-condition controls. No comparative superiority is inferred from these benchmark comparisons.

## 3. Results

### 3.1. Water Penetration Under Pressure

All standalone geopolymer variants reached the full specimen depth of 100 mm before the scheduled 72 h endpoint (Table 5). The 8 M and 10 M variants reached 100 mm after 24 h, and the 12 M variant reached 100 mm after 28 h.

**Figure 5:**
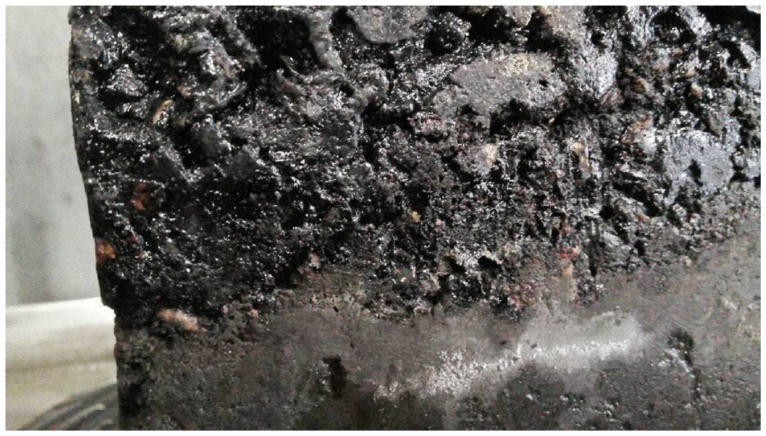
Layered specimen after the water penetration test, with the asphalt–geopolymer boundary indicated.

In the layered specimen, the geopolymer layer became wet through its full nominal 50 mm depth to the material boundary, but no leakage through the underlying asphalt layer was observed during the 72 h test (Figure 5). This observation supports functional tightness only for the tested intact system at 5 bar for 72 h; it does not demonstrate intrinsic impermeability of the geopolymer or long-term interface durability.

### 3.2. Abrasion Resistance

Three specimens were evaluated by the Böhme disc procedure. The mean volume loss was 9885.10 mm^3^/5000 mm^2^, with a range of 9729.90–10,013.90 mm^3^/5000 mm^2^ (Table 6). The values are reported as a test-specific abrasion response; they are not a same-condition comparison with a conventional overlay.

### 3.3. Thermal Properties

The transient method results are listed in Table 7. Thermal conductivity was 1.7996 W/(m·K), volumetric heat capacity was 1.5862 × 10^6^ J/(m^3^·K), and thermal diffusivity was 1.1345 × 10^−6^ m^2^/s.

For context, the literature values reported in Refs. [29,30,31,32] include a tabulated thermal conductivity of 2.0 W/(m·K) for cement concrete of similar density, 0.657–0.916 W/(m·K) for asphalt concretes with D_max_ = 12.8 mm, values up to 1.4 W/(m·K) in another asphalt source, and volumetric heat capacities of 2.7685 × 10^6^ J/(m^3^·K) for cement concrete and 2.1736 × 10^6^ J/(m^3^·K) for asphalt concrete. These contextual values were obtained under other conditions and are not same-condition controls.

### 3.4. Initial Skid Resistance

**Figure 6:**
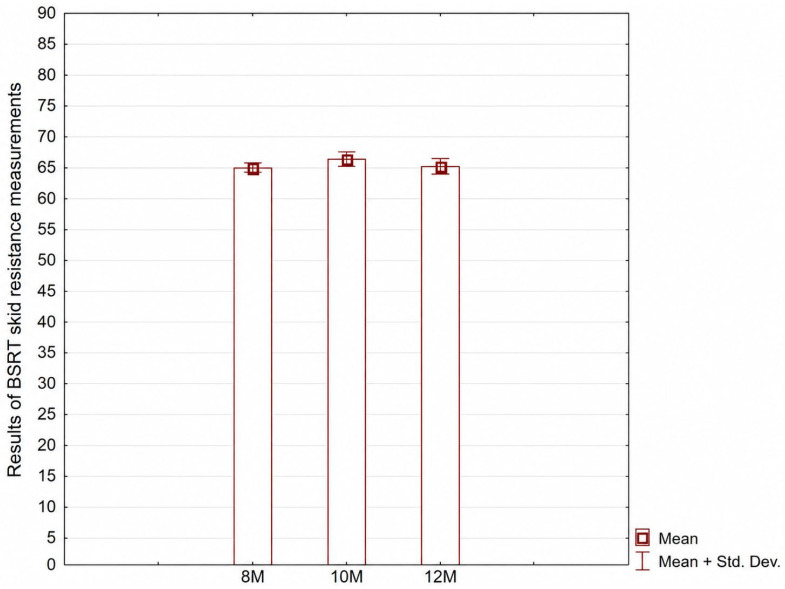
Initial BSRT results for geopolymer concretes prepared with 8 M, 10 M, and 12 M NaOH; error bars show one standard deviation.

The BSRT results are shown in Figure 6 and summarized in Table 8. The mean values were 65.0, 66.4, and 65.2 for the 8 M, 10 M, and 12 M variants, respectively. The difference among means was small, but no long-term wear sequence was performed. The results therefore characterize the initial surface response only.

According to the classification given in Ref. [3], values above 65 fall in the highest (‘good’) category, whereas values from 55 to 65 are classified as satisfactory. The mean values for the 10 M and 12 M variants were above 65; the 8 M mean was exactly 65.0 and is not described as above the boundary. This initial classification does not demonstrate retained skid resistance after polishing or prolonged trafficking.

### 3.5. Wheel Tracking Response

**Figure 7:**
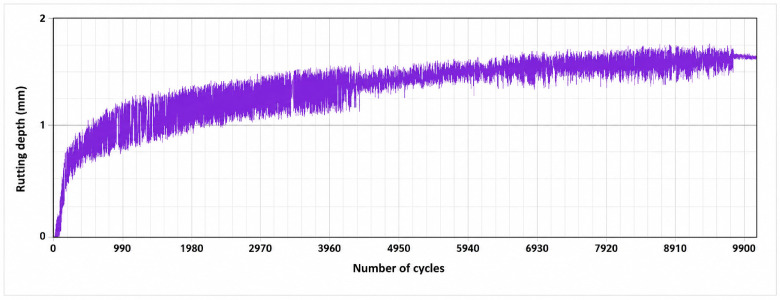
Wheel tracking curve for the asphalt–geopolymer specimen after 7 days of curing.

**Figure 8:**
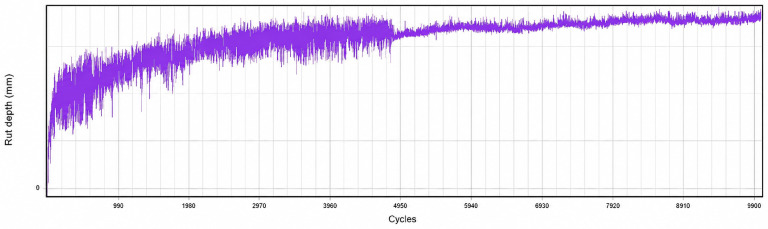
Wheel tracking curve for the asphalt–geopolymer specimen after 28 days of curing.

Figure 7 shows the wheel tracking curve after 7 days, and Figure 8 shows the corresponding curve after 28 days. The final rut depths were 1.57 and 0.72 mm, respectively. In both curves, most deformation accumulated early; the rate subsequently decreased and approached a plateau after approximately 8000 cycles.

The calculated WTSAIR values are given in Table 9. The 28-day value was approximately 29.8% lower than the 7-day value. Both were below the 0.15 mm per 10^3^ cycles benchmark cited from WT-2 [2]. This is a benchmark comparison for the tested composite, not proof of superiority over a separately tested conventional pavement layer.

### 3.6. Four-Point-Bending Fatigue

**Figure 9:**
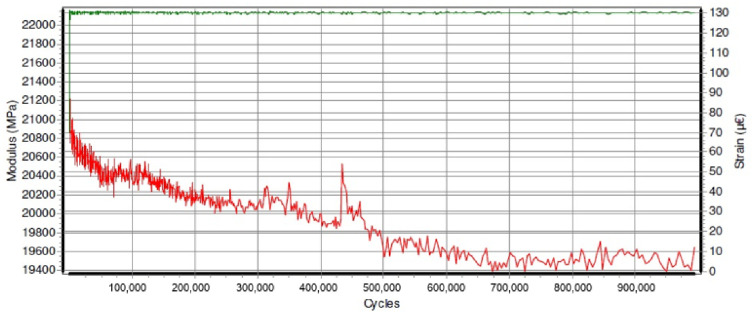
Four-point-bending response at 130 µm/m: red, stiffness modulus; green, applied strain.

**Figure 10:**
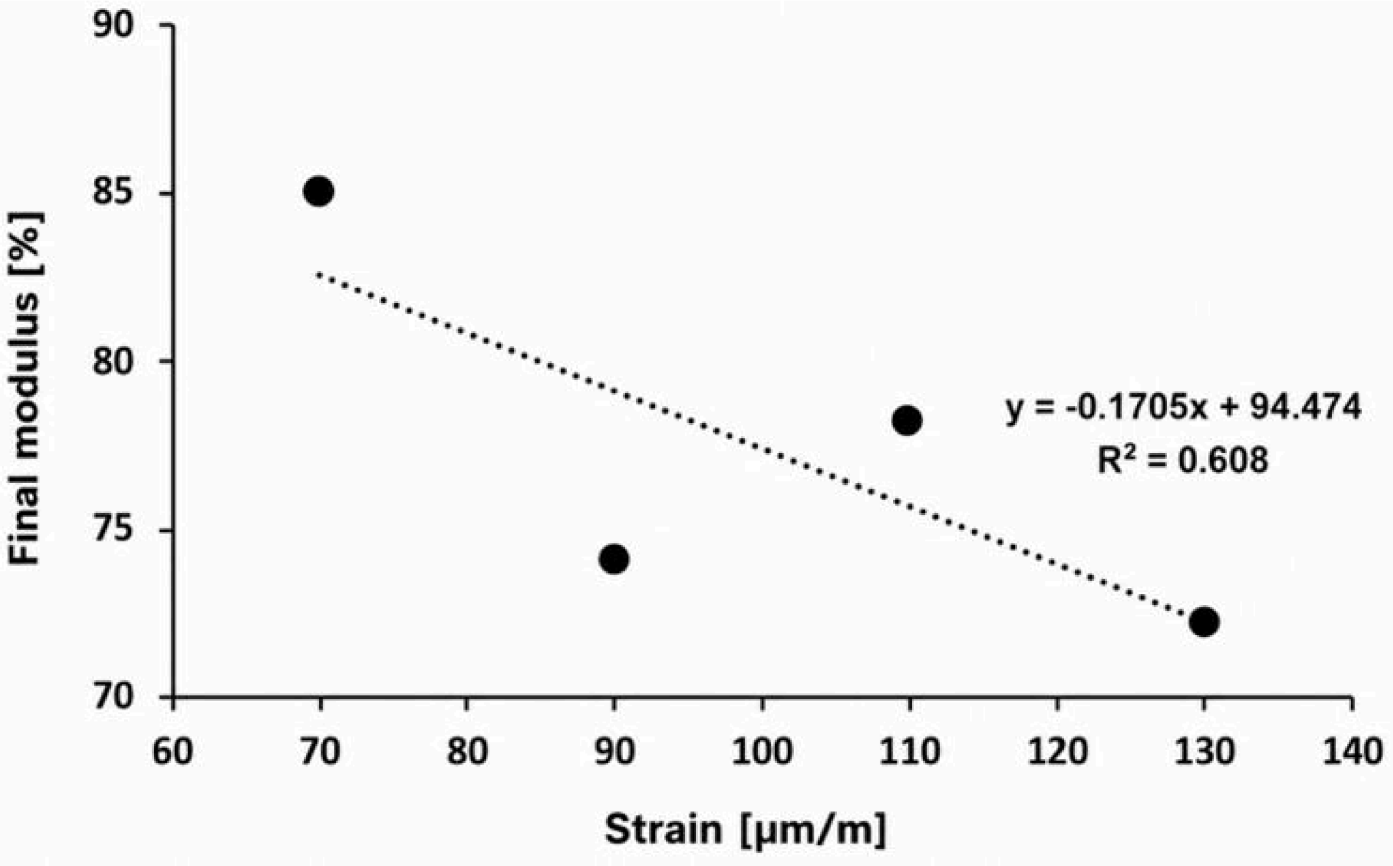
Final modulus after 10^6^ cycles; black circles: measured values; dotted line: regression.

An example 130 µm/m fatigue response is shown in Figure 9. Figure 10 and Table 10 summarize the stiffness modulus remaining after 10^6^ cycles. Across the four strain levels, 72–85% of the initial modulus remained. The 50% failure criterion cited from WT-2 [2] was not reached.

The non-monotonic 90 and 110 µm/m values are reported as measured. Because only 10 Hz was investigated, the results do not quantify performance under lower-frequency or variable-frequency traffic loading.

### 3.7. Mechanical Strength

Mechanical results for the selected formulation are summarized in Table 11. The mean flexural strength of the standalone geopolymer was 10.756 MPa, while the layered beam reached 4.482 MPa. Mean compressive strength was 61.058 MPa and mean splitting tensile strength was 3.797 MPa.

The standalone geopolymer flexural and splitting values exceeded the cited 4.5 and 3.0 MPa benchmarks for traffic categories KR1–KR4 [33]. The layered beam flexural mean was 4.482 MPa, slightly below the 4.5 MPa benchmark. These benchmark comparisons are configuration-specific and do not establish superiority over a matched conventional overlay.

### 3.8. Freeze–Thaw and De-Icing-Salt Response

Individual de-icing-salt scaling results are shown in Table 12. The mean scaled mass was 0.500 kg/m^2^ after 28 days and 0.995 kg/m^2^ after 56 days, giving m_56_/m_28_ = 1.99. The tested material met the cited FT2 criterion within this 56-day sequence; long-term salt-scaling durability was not established.

In the F150 procedure, the mean compressive strength decrease after 150 cycles was 15.878% and the mean mass loss was 3.423% (Table 13). Both values were within the 20% and 5% limits cited in PN-88/B-06250 [27] for this procedure. The result does not represent combined traffic, salt, moisture, and thermal-gradient exposure.

### 3.9. Qualitative Microstructural Observations

**Figure 11:**
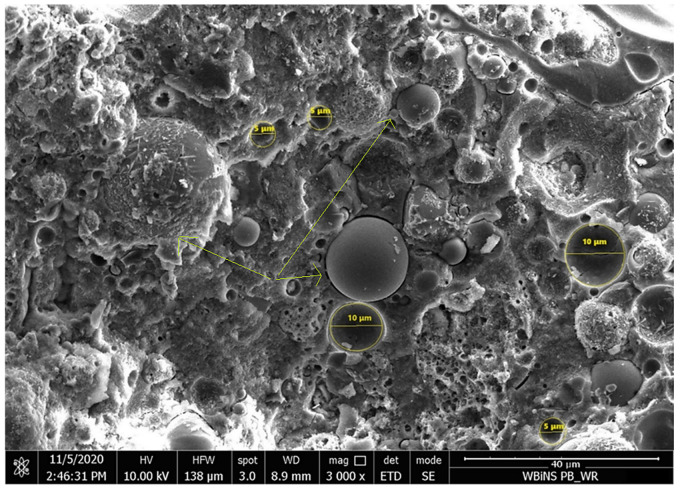
SEM image of the geopolymer matrix with unreacted fly ash particles and local pores.

Figure 11 shows the geopolymer matrix with unreacted fly ash particles and visible pores. The image demonstrates local features at the selected field of view, but it does not quantify connected porosity, pore size distribution, spacing factor, or air-void content.

**Figure 12:**
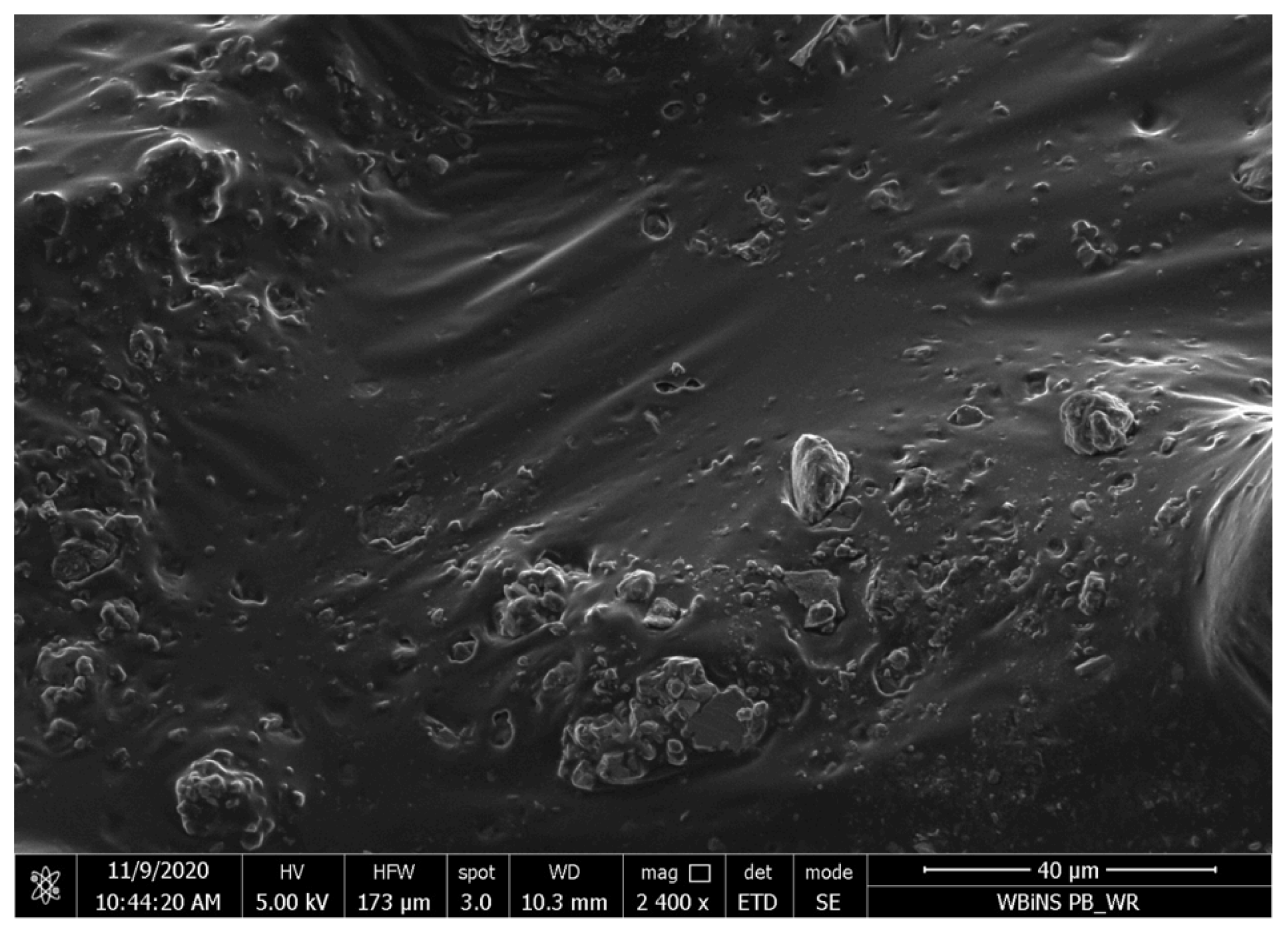
SEM image of the asphalt layer with visible limestone-filler particles.

**Figure 13:**
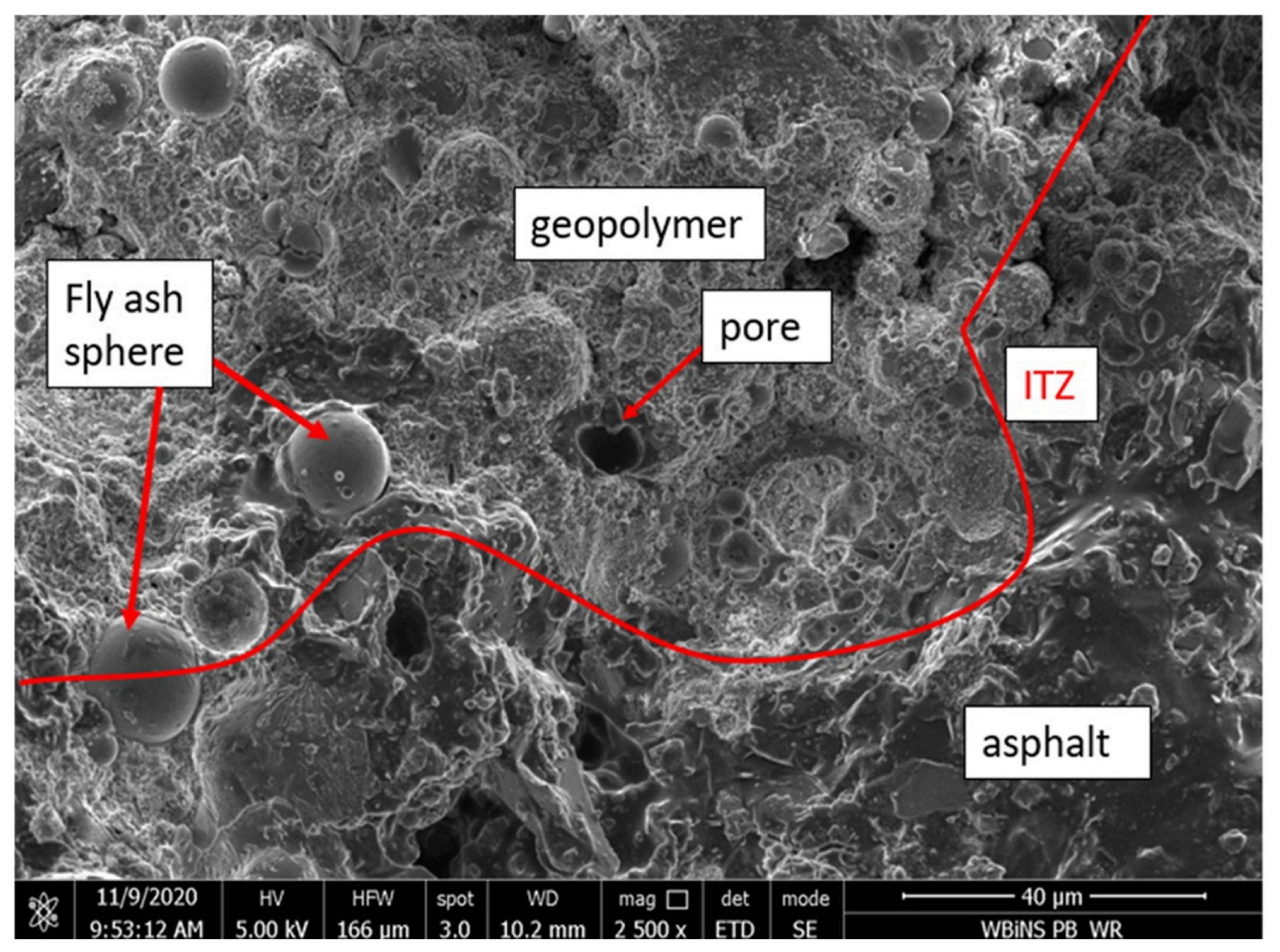
SEM image of the asphalt–geopolymer boundary with unreacted fly ash particles in the geopolymer and limestone filler in the asphalt. The red line indicates the approximate course of the asphalt-geopolymer interface, marked as the interfacial transition zone (ITZ).

Figure 12 shows the asphalt layer with limestone-filler inclusions. Figure 13 shows the material boundary at 2500× magnification. No distinct newly formed phase was identified visually at the boundary. The images show close contact and local geometric interpenetration between the layers, which is consistent with mechanical interlocking and adhesion as plausible contributors to the initial bond.

The SEM observations are qualitative. They neither establish a pore-spacing factor nor prove the mechanism responsible for freeze–thaw resistance. Quantitative image analysis, mercury-intrusion porosimetry, X-ray computed tomography, or standardized air-void measurements would be required for that purpose.

## 4. Discussion

### 4.1. Functional Performance Within the Investigated Configuration

The selected geopolymer and layered specimens produced the reported laboratory responses in mechanical strength, abrasion, initial skid resistance, wheel tracking, fatigue, and freeze–thaw tests. The 28-day wheel tracking specimen had a smaller final rut depth and lower WTSAIR than the 7-day specimen, while all fatigue beams retained more than 70% of their initial stiffness after 10^6^ cycles at the tested 10 Hz frequency. These observations apply only to the investigated materials, geometries, ages, and loading conditions.

The results do not establish universal or comparative superiority. Conventional asphalt overlays, cement concrete overlays, thin-whitetopping systems, alternative protective-layer thicknesses, and ambient- or standard-cured geopolymer controls were not tested in parallel. Comparisons with cited GDDKiA or WT-2 values indicate conformity with selected benchmarks, not dominance over alternative systems.

The BSRT results characterize the initial surface only. No polishing, accelerated wear, or long-term BSRT sequence was conducted, so retained skid resistance cannot be inferred. Likewise, the fatigue programme used a single 10 Hz frequency and did not reproduce lower-frequency, variable-amplitude, rest-period, or temperature-dependent vehicle loading.

### 4.2. Mixture Selection and Interface Evidence

The 12 M, 55% mortar-content formulation was selected by multi-criteria reasoning rather than by maximizing one response. Earlier published interlayer and dimensional-response studies [14,15] provide the formulation rationale and evidence of initial material compatibility. They are not same-condition controls for the present functional tests and do not establish bond retention after combined service exposures.

The present water penetration observations and local SEM images provide only indirect interface evidence. No direct interlayer shear, pull-off, post-fatigue bond retention, freeze–thaw-conditioned bond, or thermal-cycling delamination test was performed on the present specimens. Long-term interface durability therefore remains unverified.

### 4.3. Water Transport and the Boundary of the Tightness Claim

The water-pressure results require a two-level interpretation. At the material level, the standalone geopolymer was not intrinsically impermeable: water reached the full 100 mm depth within 24–28 h. At the system level, the intact layered specimen showed wetting through the geopolymer to the interface but no leakage was observed through the asphalt layer during 72 h at 5 bar. The supported statement is therefore functional tightness of the intact layered system only under the tested conditions.

**Figure 14:**
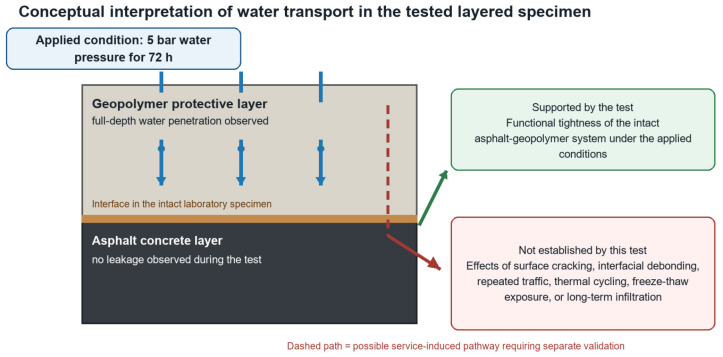
Conceptual interpretation of water transport in the tested layered specimen. Solid blue arrows represent the observed full-depth wetting of the geopolymer; the dashed red path denotes a possible service-induced pathway that was not measured.

Figure 14 distinguishes this observation from untested service scenarios. Surface cracking, interfacial debonding, fatigue damage, freeze–thaw-induced discontinuities, thermal cycling, and prolonged infiltration could create water paths that were not represented by the intact 72 h specimen.

The results identify research priorities rather than a validated waterproofing detail. Damaged and aged layered specimens, controlled surface preparation, matrix modification, interface treatment, and direct bond retention testing should be evaluated before the system is considered for service exposure.

### 4.4. Thermal Behaviour, Curing, and Field Applicability

The measured thermal conductivity, volumetric heat capacity, and diffusivity characterize the tested geopolymer. For context, the literature values reported in Refs. [29,30,31,32] were obtained for other materials and conditions and do not constitute matched controls. Without asphalt and cement concrete specimens measured in the same apparatus, or an instrumented multilayer thermal experiment, the results do not demonstrate lower pavement temperature or reduced rutting in service.

The controlled curing history is the principal constructability boundary. Preheating to 90 °C followed by passive cooling provided repeatable laboratory conditions, but conventional in situ paving cannot be assumed to reproduce that thermal history. No ambient- or standard-cured comparison series was available. Ambient curing, use of heat from freshly placed asphalt, weather sensitivity, full-scale compaction, raw material quality control, and field interface retention require separate validation.

### 4.5. Preliminary Production Cost and Environmental Context

Table 14 provides a preliminary production cost comparison for a nominal 40 mm layer. The calculated values were PLN 27.32/m^2^ for the geopolymer, PLN 32.48/m^2^ for the listed asphalt concrete, and lower values for the two listed cement concrete alternatives. The comparison does not control for structural equivalence, layer-thickness design, service interval, or construction route and therefore does not demonstrate an economic advantage.

The calculation excludes curing energy, transport, surface preparation, construction equipment, quality control, maintenance, interface repair, and end-of-life treatment. It is not a life-cycle cost analysis, and no life-cycle assessment was performed. Environmental or economic superiority is therefore not established; both require defined LCA and LCC system boundaries, validated field durability, and current process-specific data.

### 4.6. Limitations

The conclusions are subject to the following experimental and interpretive boundaries.

1. No conventional asphalt overlay, cement concrete overlay, or thin-whitetopping layer was tested as a same-condition control. No ambient-cured or standard-cured geopolymer control was included. Comparative superiority and the effect of the curing regime therefore cannot be determined.
2. Only one asphalt–geopolymer geometry was evaluated, and layer thickness was not optimized in the present programme. The tested geometry does not define an optimal field thickness or a complete construction specification.
3. The 90 °C hot-start/passive-cooling protocol is a controlled laboratory condition and limits direct transfer to conventional in situ construction.
4. The layered water-pressure test was limited to the intact condition at 5 bar for 72 h. Cracked, aged, debonded, and longer-exposure configurations were not tested, and the standalone material was not intrinsically impermeable.
5. Fatigue was tested only at 10 Hz. BSRT was measured only on the initial surface without long-term polishing or wear. De-icing-salt scaling ended at 56 days. These tests do not establish long-term fatigue, skid, or salt-scaling durability.
6. Interface durability was not verified by direct bond retention tests after fatigue, freeze–thaw exposure, thermal cycling, prolonged water exposure, or combined ageing.
7. SEM observations were qualitative and local. Mercury-intrusion porosimetry, X-ray computed tomography, quantitative image analysis, and standardized air-void analysis were not performed; spacing factor, connected porosity, and a freeze–thaw mechanism cannot be inferred from the images.
8. The production cost comparison is preliminary and is neither LCC nor evidence of economic advantage. No LCA was performed, so environmental superiority is not established.

## 5. Conclusions

The present study should be interpreted as a laboratory assessment of one selected heat-assisted configuration rather than as a field-ready pavement specification. The reported measurements support functional feasibility under the tested conditions but do not demonstrate superiority over conventional overlay systems.

1. The selected 12 M geopolymer reached mean compressive, flexural, and splitting tensile strengths of 61.058, 10.756, and 3.797 MPa, respectively. The layered beam reached 4.482 MPa in flexure, showing that standalone geopolymer strength should not be transferred directly to the composite response.
2. Wheel tracking produced final rut depths of 1.57 mm after 7 days and 0.72 mm after 28 days, with WTSAIR values of 0.020296 and 0.014238 mm per 10^3^ cycles. At the single tested frequency of 10 Hz, 72–85% of the initial stiffness modulus remained after 10^6^ cycles.
3. The mean Böhme abrasion loss was 9885.10 mm^3^/5000 mm^2^, with a maximum of 10,013.90 mm^3^/5000 mm^2^. Initial mean BSRT values were 65.0, 66.4, and 65.2 for the 8 M, 10 M, and 12 M variants; the 8 M value was exactly 65.0. These measurements do not establish long-term skid resistance retention.
4. Mean de-icing-salt scaling was 0.500 kg/m^2^ after 28 days and 0.995 kg/m^2^ after 56 days. After 150 freeze–thaw cycles, mean compressive strength decrease and mass loss were 15.878% and 3.423%, respectively. Exposure beyond 56 days was not conducted for the scaling series.
5. Full-depth water penetration in the standalone geopolymer precludes a claim of intrinsic impermeability. The absence of leakage through the asphalt layer in the intact composite supports functional tightness only for the tested 5 bar, 72 h condition and does not establish long-term interface durability.
6. The thermal results characterize the tested material, while the qualitative SEM images provide local morphological observations only. Neither dataset demonstrates a service-temperature benefit, quantitative pore structure, or a freeze–thaw mechanism.
7. Because no ambient-cured or same-condition control overlay was tested, only one composite geometry was examined, and direct bond retention, long-term skid and salt-scaling durability, LCA, and LCC remain unavailable, the findings define application boundaries rather than implementation criteria. Further validation is required before extending the results to conventional in situ paving.

## Figures and Tables

**Table 1:** Chemical composition of the fly ash.

Constituent or Property	Result (%)
SiO_2_	54.60
Al_2_O_3_	25.30
MgO	1.80
CaO	2.14
Na_2_O-equivalent alkalis	2.68
Na_2_O	0.84
K_2_O	2.80
Components insoluble in Na_2_CO_3_	>40
P_2_O_5_	6.69
SO_3_	0.35
Sulfides	<0.01
Reactive CaO	1.84
Reactive SiO_2_	42.36
Free CaO	<0.03
Loss on ignition	4.37

**Table 2:** Composition of AC16W 35/50 asphalt concrete.

Component	Mass Fraction (%)
Limestone filler	4.77
0/2 mm glacial sand	17.17
2/5 mm crushed glacial gravel	24.80
5/11 mm crushed glacial gravel	20.03
11/16 mm crushed glacial gravel	28.62
35/50 paving bitumen	4.60

**Table 3:** Reference geopolymer concrete composition.

Component	Content (kg/m^3^)
Alkaline activator	200
Fly ash	400
Sand 0/2 mm	725
Basalt aggregate 2/5 mm	327
Basalt aggregate 5/8 mm	368
Melaphyre aggregate 8/11 mm	397

**Table 4:** Test programme for the geopolymer and asphalt–geopolymer specimens.

Property	Standard or Procedure	Specimen	Reported Response and Key Condition
Water penetration under pressure	PN-EN 12390-8:2011 [20]	100 mm geopolymer cubes and layered cubes	Penetration depth; 5 bar; 72 h
Abrasion	PN-EN 1338:2005 [21]	Specimens cut from cubes	Böhme volume loss
Initial skid resistance	PN-EN 13036-4:2011 [22]	300 × 400 × 100 mm slabs	BSRT index
Wheel tracking	PN-EN 12697-22:2020 [23]	300 × 400 × 100 mm layered slabs	Rut depth and WTSAIR; 65 °C in air
Fatigue	PN-EN 12697-24:2018 [24]	55 × 55 × 400 mm beams	Stiffness retention; 10 Hz; 70–130 µm/m; 10^6^ cycles
Flexural strength	PN-EN 12390-5:2019 [25]	100 × 100 × 500 mm beams	Mean flexural strength
Splitting tensile strength	PN-EN 12390-6:2011 [26]	100 mm cubes	Mean splitting tensile strength
Standard freeze–thaw	PN-88/B-06250 [27]	100 mm cubes	Mass loss and compressive strength decrease after 150 cycles
De-icing-salt freeze–thaw scaling	PKN-CEN/TS 12390-9:2017-07 [28]	Cut cube specimens	Scaled mass after 28 and 56 days
Thermal properties	Transient method, ISOMET 2114 (APPLIED PRECISION s.r.o., Bratislava, Slovakia)	Geopolymer specimens	Thermal conductivity, volumetric heat capacity, thermal diffusivity
Microstructure	SEM	Geopolymer, asphalt, and interface fragments	Qualitative morphology only

**Table 5:** Water penetration under 5 bar pressure in standalone geopolymer specimens.

NaOH Concentration	Penetration Depth (mm)	Time at Which 100 mm Was Reached
8 M	100	24 h
10 M	100	24 h
12 M	100	28 h

**Table 6:** Summary of Böhme disc abrasion results.

n	Mean (mm^3^/5000 mm^2^)	Minimum	Maximum	Standard Deviation
3	9885.10	9729.90	10,013.90	143.83

**Table 7:** Thermal properties of the geopolymer concrete.

Parameter	Symbol	Value	Unit
Thermal conductivity	λ	1.7996	W/(m·K)
Volumetric heat capacity	cρ	1.5862 × 10^6^	J/(m^3^·K)
Thermal diffusivity	a	1.1345 × 10^−6^	m^2^/s

**Table 8:** Summary of initial BSRT measurements.

NaOH Concentration	n	Mean	Minimum	Maximum	Standard Deviation
8 M	5	65.0	64	66	0.707
10 M	5	66.4	65	68	1.140
12 M	5	65.2	63	66	1.304

**Table 9:** Wheel tracking results.

Test Age (Days)	Rut Depth at 5000 Cycles (mm)	Rut Depth at 10,000 Cycles (mm)	WTSAIR (mm per 10^3^ Cycles)
7	1.4643	1.56578	0.020296
28	0.64913	0.72032	0.014238

**Table 10:** Stiffness modulus retention after 10^6^ cycles at 10 Hz.

Applied Strain (µm/m)	Final Modulus as Percentage of Initial Modulus (%)
70	85
90	74
110	78
130	72

**Table 11:** Mechanical strength summary.

Property and Specimen	n	Mean (MPa)	Minimum	Maximum	Standard Deviation
Flexural strength, geopolymer beam	5	10.756	9.870	12.160	0.931
Flexural strength, layered beam	5	4.482	4.380	4.710	0.139
Compressive strength, geopolymer	5	61.058	59.400	62.800	1.455
Splitting tensile strength, geopolymer	5	3.797	3.649	3.924	0.123

**Table 12:** De-icing-salt scaling results.

Specimen	m_28_ (kg/m^2^)	m_56_ (kg/m^2^)
1	0.61	1.22
2	0.41	0.80
3	0.58	1.12
4	0.40	0.84
Mean	0.500	0.995

**Table 13:** Summary of the F150 freeze–thaw response.

Response	n	Mean (%)	Minimum	Maximum	Standard Deviation	Cited Limit (%)
Compressive strength decrease	6	15.878	14.286	17.148	1.020	20
Mass loss	6	3.423	2.818	4.097	0.453	5

**Table 14:** Preliminary production cost comparison for a nominal 40 mm layer.

Net Production Cost	Asphalt Concrete ACS11S	Pavement Concrete C30/37 XF4	Roller-Compacted Concrete	Geopolymer Concrete
PLN/t	350	176	118	276
PLN/m^3^	812	415	260	683
PLN/m^2^ for a 40 mm layer	32.48	16.60	10.40	27.32

## Data Availability

The original contributions presented in this study are included in the article. Further inquiries can be directed to the corresponding author.
